# Assessment of Gastric Remnant Activity, Symptoms, and Quality of Life Following Gastric Bypass

**DOI:** 10.1007/s11695-024-07534-5

**Published:** 2024-10-13

**Authors:** Tim Hsu-Han Wang, Chris Varghese, Stefan Calder, Armen A. Gharibans, Nicholas Evennett, Grant Beban, Gabriel Schamberg, Greg O’Grady

**Affiliations:** 1 Department of Surgery, University of Auckland, Auckland, New Zealand; 2 Alimetry (New Zealand), Auckland, New Zealand; 3 Department of General Surgery, Auckland City Hospital, Auckland, New Zealand

**Keywords:** Gastric bypass, Gastric emptying, Gastric myoelectrical activity, High-resolution electrogastrography, Slow waves, Gastric Alimetry

## Abstract

**Introduction:**

While most gastric bypass patients recover well, some experience long-term complications, including nausea, abdominal pain, food intolerance, and dumping. This study aimed to evaluate symptoms and quality of life (QoL) in association with the residual activity of the remnant stomach.

**Methods:**

Patients undergoing gastric bypass and conversion-to-bypass were recruited. The Gastric Alimetry® System (Auckland, NZ) was employed, comprising a high-resolution electrode array, wearable reader, and validated symptom logging app. The protocol comprised 30-min fasting baseline, a 218-kCal meal stimulus, and 4-h of post-prandial recordings. Symptoms and QoL were evaluated using validated questionnaires. Remnant gastric electrophysiology evaluation included frequency, BMI-adjusted amplitude, and Gastric Alimetry Rhythm Index (GA-RI, reflecting pacemaker stability), with comparison to validated reference intervals and matched controls.

**Results:**

Thirty-eight participants were recruited with mean time from bypass 46.8 ± 28.6 months. One-third of patients showed moderate to severe post-prandial symptoms, with patients’ median PAGI-SYM 28 ± 19 vs controls 9 ± 17 (*p* < 0.01); PAGI-QOL 37 ± 31 vs 135 ± 22 (*p* < 0.0001). Remnant gastric function was markedly degraded shown by undetectable frequencies in 84% (vs 0% in controls) and low GA-RI (0.18 ± 0.08 vs 0.51 ± 0.22 in controls; *p* < 0.0001; reference range > 0.25). Impaired GA-RI and amplitude were correlated with worse PAGI-SYM and PAGI-QOL scores.

**Conclusion:**

One-third of post-bypass patients suffered significant upper GI symptoms with reduced QoL. The bypassed remnant stomach shows highly deranged electrophysiology in-situ, reflecting disuse degeneration. These derangements correlated with QoL; however, causality is not implied by the present study.

**Supplementary Information:**

The online version contains supplementary material available at 10.1007/s11695-024-07534-5.

## Introduction

Gastric bypass is a common procedure that is predominantly performed for obesity, with a long track record of safety and efficacy [1]. Sleeve gastrectomy is an alternative procedure, which sometimes may be converted to bypass in the event of intractable complications such as reflux, hiatus hernia and strictures [2].

While varying techniques and terminology for bypass exists (e.g., Roux-en-Y, single-anastomosis, mini-loop, omega-loop), all involve the formation of small gastric pouch, connecting the esophagus with the small intestine, and a gastric remnant that is excluded from digestive continuity. The majority of patients do well after these bariatric procedures, however, a proportion experience long-term complication, including nausea, abdominal pain, anastomotic stricture, and dumping syndromes [2–4]. These can significantly impact quality of life, with evidence suggesting variable return to baseline quality of life dependent on the procedure performed and weight change achieved [5–8].

While there are several studies evaluating symptoms and quality of life after bypass surgery [5–8], meal associations with symptoms and the underlying gastric remnant motor function have not been characterized in detail. We hypothesize the remnant stomach in its dormancy will become atrophic with the diversion of contents; however, the stomach likely also remains bioelectrically and neurally active, which could be relevant to symptom associations [9, 10]. Gastric dysrhythmias are of interest as they have been implicated in chronic nausea [11–13], as well as post-surgical symptoms after sleeve gastrectomy, esophagectomy, and partial gastrectomy [14–16], albeit in patients with stomachs in continuity.

Gastric Alimetry® (Alimetry, New Zealand) is a new non-invasive test to evaluate gastric electrophysiology and function at high resolution (HR)[13]. The test is highly validated and recently receiving regulatory approvals for clinical use [9, 15, 17]. It also includes a validated evaluation of patient-reported symptom evolution in relation to a meal, using an App, which correlates to quality of life [18]. The aim of this study was therefore to assess the symptoms and quality of life (QoL) of patients following gastric bypass and conversion to bypass, in addition to remnant gastric function, using the new Gastric Alimetry system.

## Methods

### Patient Population

Consecutive patients who underwent either a gastric bypass or a sleeve gastrectomy with subsequent conversion to gastric bypass performed at Auckland City Hospital (Auckland, New Zealand) between 2013 and 2022 were recruited. Ethics approval was obtained from the Auckland Health Research Ethics Committee (AH1125) and informed consent was obtained from all patients. Patients who had a history of skin allergy, evidence of mechanical gastric, or small bowel obstruction as a cause for their symptoms, BMI > 40 (excluded due effect of abdominal wall thickness on signal attenuation [19, 20]) and those with insulin-dependent diabetes were excluded.

Clinical data including operation notes, imaging, endoscopy, and histopathology (if applicable) were evaluated. All gastric bypass procedures involved a peri-gastric dissection to preserve vagus nerve function to the distal stomach, and no patients had a prior fundoplication. Patients were also followed up within 1 week for adverse reactions. A healthy control cohort was also recruited, who had a similar caloric content meal, being matched based on patient gender, age, and BMI.

### Quality of Life Assessments

At the time of Gastric Alimetry testing, validated questionnaires were employed including the PAGI-SYM, PAGI-QOL and EQ-5D-5L which assesses the patients’ symptoms and quality of life over the preceding 2 weeks [21–23]. Individual and total scores were obtained and analyzed.

### Gastric Alimetry

Gastric myoelectrical activity coordinates gastric motility, with an intrinsic pacemaker rhythm generated and propagated by the interstitial cells of Cajal (ICC)[10]. The normal pacemaker site is located on the greater curvature of the upper corpus, while the fundus (and therefore gastric bypass pouch) is generally electrical quiescent [24] The small intestine operates within a different frequency range, which can therefore be readily distinguished from gastric signals [25].

**Fig. 1:**
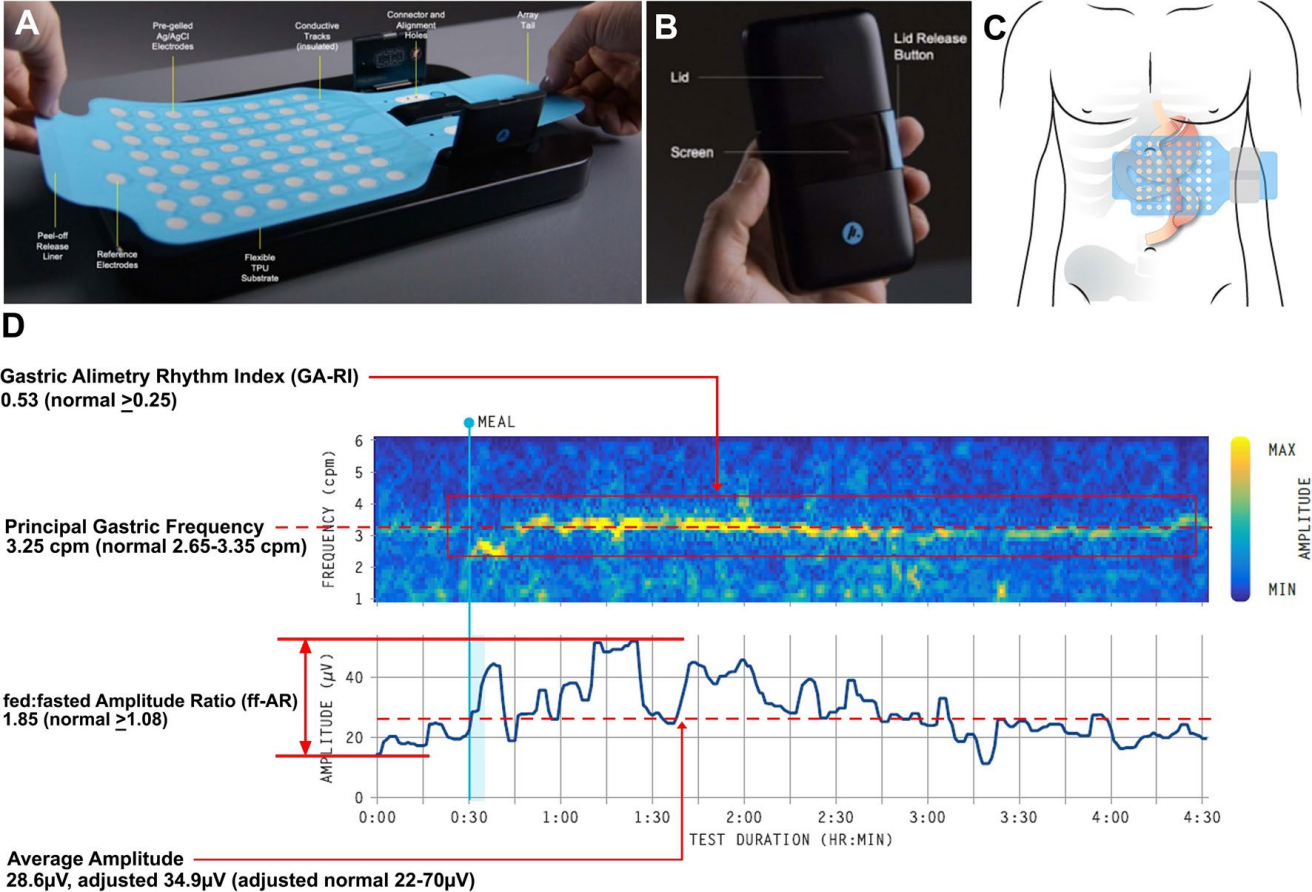
**A** Photographic representation of the Gastric Alimetry array. **B** Photograph of the Gastric Alimetry reader. **C** Representative diagram of the gastric bypass and its relation to array placement. **D** Example matched control case with specific reference to the validated normal range

Gastric Alimetry was performed to measure the gastric myoelectrical activity under a protocol adapted for patients with a reduced gastric volume. This device comprises an HR stretchable electrode array (8 × 8 electrodes; 20-mm spacing; 196 cm^2^), a wearable Reader, validated iOS app for symptom logging, and a cloud-based reporting platform (Fig. 1A, B) [18, 26, 27]. A complete description of the device, its principles of operations, validation of specificity for gastric signals, and its clinical applications are presented elsewhere [13, 26, 28, 29]. Baseline recordings were performed in the first 30 min, followed by a 218-kCal meal, comprising of 100 mL of Ensure (93 kCal; Abbott Nutrition, IL, USA) and half an oatmeal energy bar (125 kcal, 2.5 g fat, 22.5 g carbohydrate, 5 g protein, 3.5 g fiber; Clif Bar & Company, CA, USA) to account for the partial loss of stomach reservoir, consumed over 10 min and a 4-h post-prandial recording in order to capture a full gastric activity cycle. Patients were seated in a reclined chair and were asked to limit movement, talking, and sleeping, but were able to read, watch media, work on a mobile device, and mobilize for comfort breaks. Symptom development or changes were recorded on the Gastric Alimetry App, calculated into a Total Symptom Burden Score as defined and validated by Sebaratnam et al. [18].

Spectral (frequency-amplitude) analysis was performed, encompassing four established metrics derived from the Gastric Alimetry system [30]: Principal Gastric Frequency (PGF), BMI-Adjusted Amplitude, Gastric Alimetry Rhythm Index (GA-RI; reflecting gastric pacemaker activity and stability), fed:fasted amplitude ratio (ff-AR; indicating meal response with contractions), with comparison to reference intervals and the matched control cohort separately [20]. Frequency was not reported if there was no stable rhythm (as automatically determined in the Gastric Alimetry Report) [27]. Adverse events were recorded.

### Data Analyses

Statistical analysis was performed using GraphPad Prism v.8 (San Diego, CA, USA) and R v.4.0.1 (R Foundation for Statistical Computing, Vienna, Austria). Analyses were performed on bypass-only cohort, conversion to bypass-only cohort and a combined cohort (i.e., bypass-only and conversion to bypass group). Symptom and quality of life comparisons with the matched healthy controls were performed using the unpaired Student’s *t*-test. Correlation analysis between spectral metrics, total symptom burden score, and QoL scores were performed with Spearman’s correlation. All data is presented as mean ± SD unless otherwise stated, with *p* < 0.05 considered as statistically significant.

## Results

### Patient Population

Twenty patients were recruited in total: 13 bypasses, 6 sleeve converted to bypass, 1 resectional gastric bypass (8 males, 12 females; median age 55.5 years old; age range 33–68 years old; median BMI 30.1; BMI range 21–38.8). Indication for all bypasses were for obesity. Indications for the sleeve converted to bypass included reflux and hiatus hernia (*n* = 5) and narrowing of post-sleeve gastrectomy stomach and twisting (*n* = 1). The resectional gastric bypass was a patient who developed intermittent gastric distention following fundoplication without any evidence of a mechanical cause, which was included as a negative control showing the absence of gastric activity following a meal challenge (results in Supplementary Fig. 1). All other reconstructions were performed with a Roux-en-Y reconstruction and no gastric resection. Median time from surgery was 43.5 months (range 4–107 months). Patient and matched control demographics are presented in Table 1, showing no differences in BMI, age, or gender between groups.

**Table 1:** Patient and match control demographic, quality of life (QOL) and spectral characteristics data. The *p* values in bold denote statistically significant difference between the groups. *TSBS*, Total Symptom Burden Score; *PAGI-SYM*, Patient Assessment of Upper Gastrointestinal Symptom Severity Index; *PAGI-QOL*, Patient Assessment of Upper GastroIntestinal Disorders-Quality of Life; *EQ-5D-5L*, questionnaire developed by the EuroQol Group; *ff-AR*, fed:fasted amplitude ratio; *GA-RI*, Gastric Alimetry Rhythm Index

Characteristics	Patients (*n* = 19)	Matched controls (*n* = 19)	*p* value
Age	55.0 ± 9.5	55.4 ± 10.2	0.90
BMI (kg/m^2^)	29.5 ± 4.5	28.8 ± 10.5	0.78
Gender			
Male	7	8	1.00
Female	12	11	
Duration since surgery (months)	46.8 ± 28.6	N/A	
QoL			
TSBS	6.7 ± 9.13	0.7 ± 2.36	0.01
PAGI-SYM	28 ± 19	9 ± 17	< 0.01
PAGI-QOL	37 ± 31	135 ± 22	< 0.0001
EQ-5D-5L	0.83 ± 0.22	0.95 ± 0.08	0.04
Self-reported EQ-5D-5L	71 ± 17	89 ± 6	0.0005
Spectral Characteristics			
Frequency (2.65–3.35 cpm)	3.37 ± 0.1 (n = 3)	3.08 ± 1.45	0.02
BMI-Adjusted amplitude (22–70 μV)	24.8 ± 5.2	36.1 ± 14.0	< 0.0001
ff-AR (≥ 1.08)	1.30 ± 0.22	2.06 ± 1.05	0.01
GA-RI (≥ 0.25)	0.18 ± 0.08	0.51 ± 0.22	< 0.0001

### Symptom Burden and Quality of Life

One-third of post-bypass patients were found to have moderate to severe post-prandial symptoms. Differences in the total symptom burden score, upper gastrointestinal symptoms and QoL scores between patients and controls are reported in Table 1, with patients showing substantially worse scores across all tests, including time-of-meal symptoms and longer-term questionnaires. Comparisons in QoL data and total symptom burden score for the different bypass groups are presented in Table 2. The conversion to bypass group had a lower average QoL compared to the bypass-only group; however, this did not reach statistical significance within the present cohort size.

**Table 2:** Total Symptom Burden Score (TSBS) and Quality of life (QoL) data on the bypass cohort, conversion to bypass cohort, and combined bypass and conversion to bypass cohort. *PAGI-SYM*, Patient Assessment of Upper Gastrointestinal Symptom Severity Index; *PAGI-QOL*, Patient Assessment of Upper GastroIntestinal Disorders-Quality of Life; *EQ-5D-5L*, questionnaire developed by the EuroQol Group

QOL	Bypass (*n* = 13)	Conversions (*n* = 6)	*p* value	Overall (*n* = 19)
TSBS	3.8 ± 3.4	12.9 ± 14.3	0.18	6.7 ± 9.13
PAGI-SYM	22 ± 11	41 ± 26	0.14	28 ± 19
PAGI-QOL	30 ± 24	52 ± 40	0.26	37 ± 31
EQ-5D-5L	0.85 ± 0.24	0.80 ± 0.19	0.67	0.83 ± 0.22
Self-reported EQ-5D-5L	74 ± 16	65 ± 19	0.30	71 ± 17

### Gastric Spectral Data

Eighteen out of nineteen participating patients had at least 1 abnormal gastric slow wave spectral abnormality in the remnant stomach, including 17 patients who had 2 or more abnormalities compared to the test reference intervals [20]. Data for all spectral metrics by group are presented in Table 3. Due to the very low GA-RI value for almost all patients (17/19), the principal gastric frequency values could only be identified in 3 subjects (2 of the 3 having normal GA-RI values). By contrast, a principal gastric frequency was reliably detected in all controls, including during their fasting periods. All 3 cases with a preserved PGF were in the bypass-only group, with an average time since bypass of 38.8 ± 25.1 months. The GA-RI value was lower in the conversion-only group vs. the bypass-only group, although this did not reach statistical significance in the sample size (0.020 ± 0.08 vs 0.13 ± 0.06; *p* = 0.07) (Table 3).

**Table 3:** Gastric slow wave spectral characteristics data on the bypass cohort, conversion to bypass cohort, and combined bypass and conversion to bypass cohort. Note that the frequency for all cohort only accounted for a total of 3 cases with the remainder had rhythms too unstable for a frequency to be ascertained therefore a suitable frequency was not calculated. The overall frequency presented is therefore unreliable. The bold numbers denote abnormal results compared to the validated reference range. *TSBS*, Total Symptom Body Score; *ff-AR*, fed:fasted amplitude ratio; *GA-RI*, Gastric Alimetry Rhythm Index

Spectral characteristics	Bypass (*n* = 13)	Conversions (*n* = 6)	*p* value	Overall (*n* = 19)
Frequency (2.65–3.35)	N/A	N/A	N/A	3.37 ± 0.1
BMI-Adjusted amplitude (22–70)	25.0 ± 3.8	24.3 ± 8.4	0.86	24.8 ± 5.2
ff-AR (≥ 1.08)	1.28 ± 0.22	1.34 ± 0.22	0.57	1.30 ± 0.22
GA-RI (≥ 0.25)	0.20 ± 0.08	0.13 ± 0.06	0.07	0.18 ± 0.08
TSBS (Symptom Score ≥ 3)	62% (8/13)	83% (5/6)	-	68% (13/19)

**Fig. 2:**
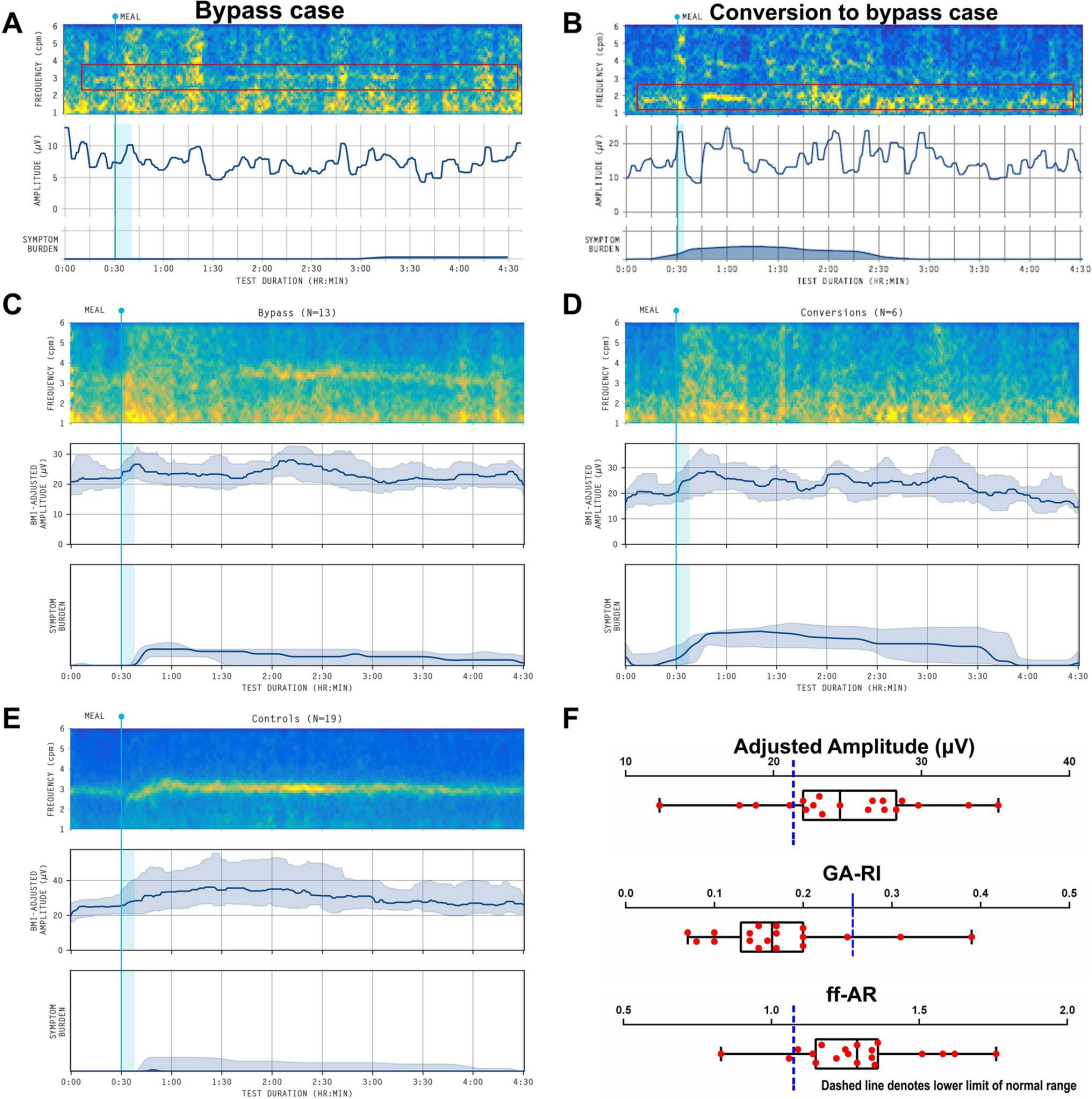
Example spectral diagram of cases from **A** bypass cohort, **B** conversion to gastric bypass cohort. Boxed red line in **A**, **B** indicates the degraded principal gastric frequency, however, these are significantly lower in amplitude than healthy patients. **C**, **D**, **E** are average spectrogram with median (IQR shaded) BMI-adjusted amplitude and symptom burden for bypass cohort (**C**), conversion to bypass cohort (**D**), and matched control cohort (**E**). **F** Box and whisker diagram of the combined bypass and conversion to bypass cases. Dashed blue line denotes the lower limit of normal range. Note that no box and whisker is available for the principal gastric frequency as there were only 3 cases where this value was able to be determined

A reference spectrogram of a healthy control is presented in Fig. 1D, together with the Gastric Alimetry test reference intervals. Example spectrograms from a bypass case and conversion to bypass case, with highly degraded gastric activity by comparison, are shown in Fig. 2A and B respectively. Summary spectrograms (combining all subjects’ data) are shown for the bypass-only group, conversion to bypass group, and controls, in Fig. 2C–E, again showing dramatically degraded gastric activity following bypass. Comparisons to reference intervals are depicted in Fig. 2F, with the main abnormality being reduced GA-RI.

### Correlation Analysis

**Fig. 3:**
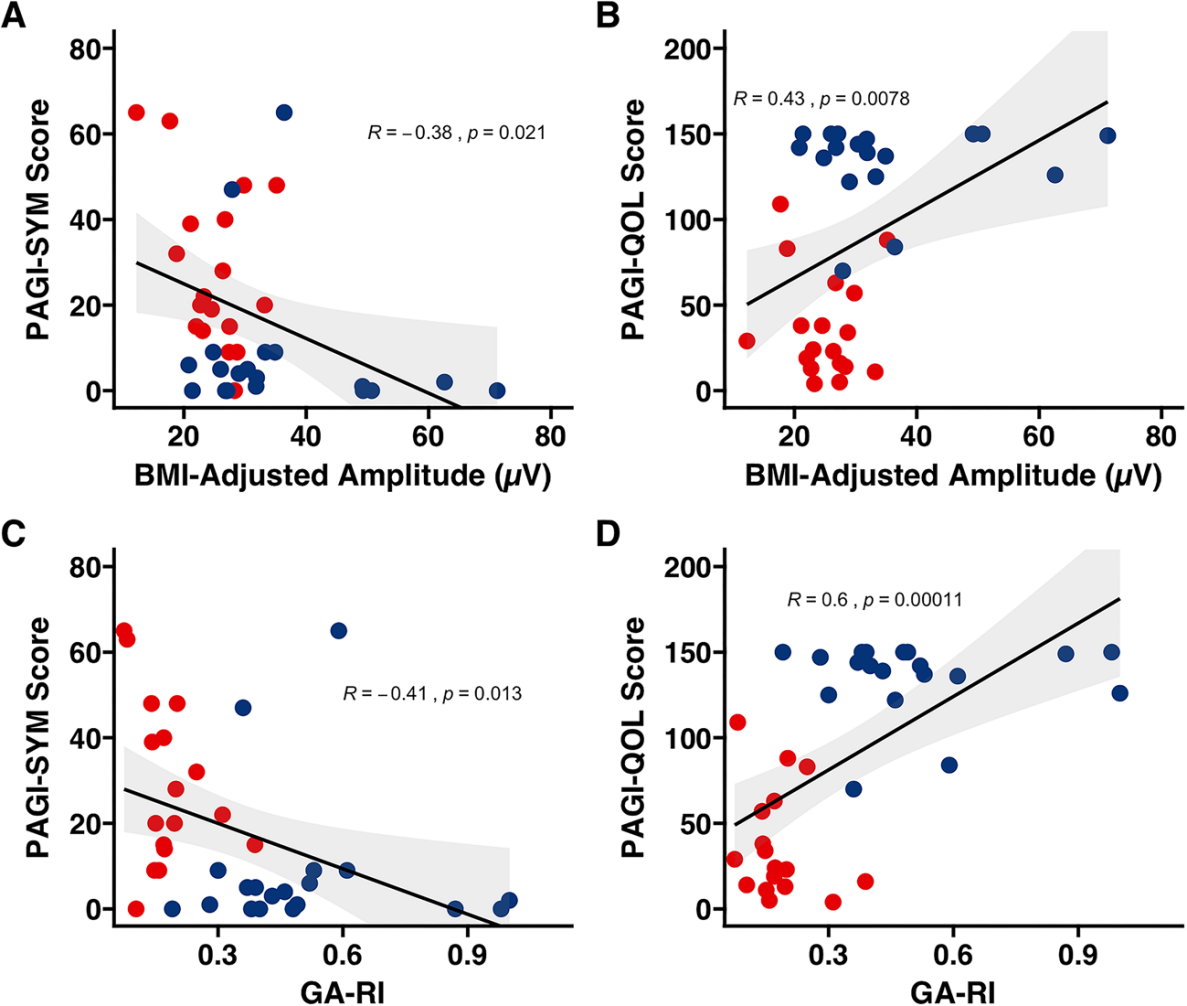
Scatter plot between BMI-adjusted amplitude with PAGI-SYM and PAGI-QOL scores and GA-RI with PAGI-SYM and PAGI-QOL scores. Red dots indicate the patient cohort while the blue dots indicate the matched control cohort. The shaded gray area indicates a 95% confidence interval. Individual trend lines are also drawn for the entire cohort with *R* value and *p* value presented. GA-RI, Gastric Alimetry Rhythm Index

Higher PGF (*r* =  − 0.57), lower ff-AR (*r* = 0.42), lower GA-RI (*r* = 0.63), and lower BMI-adjusted amplitude (*r* =  − 0.43) were all associated with lower quality of life as measured by the PAGI-QOL score (*p* < 0.05). Similarly, lower GA-RI (*r* =  − 0.41), and lower BMI-adjusted amplitude (*r* =  − 0.38) were also significantly associated with higher symptom burden as measured by PAGI-SYM score (*p* < 0.05). Correlation plots for BMI-adjusted amplitude and GA-RI are shown in Fig. 3. For specific symptoms, PGF was found to be correlated with early satiety, BMI-adjusted amplitude was correlated with nausea and vomiting, and ff-AR was correlated with early satiety and symptoms of heartburn and regurgitation (*p* < 0.05).

### Safety

No adverse events arising from the Gastric Alimetry test were identified in any subject.

## Discussion

Index and converted gastric bypass surgery involve reconstruction of the gastrointestinal tract with exclusion of a gastric remnant. Postoperative symptoms can be persistent, including nausea, vomiting, pain and dumping; however, the pathophysiology may be unclear with normal endoscopy or imaging [2]. The results of this study show that over one-third of patients experienced moderate to severe symptoms with worse QoL compared to matched controls. Remnant gastric stomach function was also found to be highly degraded in the patient cohort, with these changes found to be correlated to worse symptom burdens and lower quality of life scores.

The associations between remnant stomach degeneration, foregut symptoms, and quality of life in this study are novel. Degradation of the gastric pacemaker system in the bypassed stomach is expected due to disuse degeneration, as pacemaker system degeneration has also been described in animal stomachs after marked calorie restriction, thought to be mediated by reduced local production of IGF-1 [31]. Gastric bypass in itself could also conceivably modify the gastric electrical conduction system through disconnection of the stomach and partial denervation [32]. It is plausible that the gastric dysrhythmias could contribute to upper gastrointestinal symptoms in bypass patients, as post-surgical gastric dysrhythmias have been linked to symptoms in previous postoperative populations [14, 15, 33, 34].

The GA-RI was reduced in the conversion group compared to the bypass-only group, which neared significance (*p* = 0.07) despite the small number of conversion patients in this subgroup (*n* = 6). This is likely a true finding, reflecting the effect of the previous vertical sleeve gastrectomy, which definitively removes the native pacemaker and will have already caused underlying gastric rhythm disturbances [14]. The adjusted amplitude and the ff-AR reductions compared to the matched control cohort are explained by pacemaker degeneration, smooth muscle atrophy, a reduced volume of electrically active functioning gastric tissue, and the lack of food content stimulating the gastric remnant [32].

While there have been previous studies assessing the effects of surgical techniques on the postoperative outcomes and quality of life in bypass, along with manometric assessments of the gastric pouch and the Roux limb, studies of the remnant gastric conduction system have been sparse prior to this study [35, 36]. This is likely due to the lack of adequately high-resolution techniques to assess the electrophysiology of the stomach. Recent technological advances in the form of invasive and non-invasive high-resolution gastric mapping techniques have now significantly improved our understanding of gastric electrophysiology in health and disease [9, 24, 37]. Legacy techniques including electrogastrography (EGG) have previously been attempted to assess the electrical activity of the stomach following surgery; however, this is limited by low resolution and high sensitivity to noise. Gastric Alimetry overcomes these problems by employing an HR array together with sophisticated signal processing algorithms [26, 27], to non-invasively assess the electrophysiology of the gastric remnant and to also simultaneously record symptom development and progression. Previously, Gastric Alimetry has been exclusively performed in patients with normal gastric anatomies, in whom reference intervals were developed [20, 30, 37]. As this study now involves a modified Gastric Alimetry protocol to account for the effect of surgery on the stomach, the authors used both the validated reference intervals and a cohort of matched control patients (with a similar calorie load meal challenge) as a comparison.

The strengths of this study include the recruitment of symptomatic and asymptomatic patients in the patient population, and the use of a matched control cohort for comparison. The recruitment of the patient who had a resectional gastric bypass (which is rare) also allowed the confirmation that all the data captured in this study arose from the gastric remnant, as there was minimal post-meal activity identified in the gastric pouch (former fundus), which is an area of the stomach known to be electrically quiescent [10, 38]. The main limitation of this study was its dominant focus on remnant gastric motility, such that other potential sources of symptoms such as pouch and Roux limb function, together with other contributing disorders, were not systematically evaluated. A causal association should not be inferred from our results, particularly because the remnant stomach is excluded from GI continuity and symptoms had a post-prandial character. Therefore, it is highly likely that symptom genesis post-bypass is multi-factorial, with other contributing sources including gastric pouch stretch, Roux stasis syndrome, and post-surgical sequelae [32, 36]. Future work could now focus on addressing the causality of the novel finding that degradations of gastric electrophysiology correlated with symptoms, which could be approached using frameworks such as the Plausibility Criteria proposed by Tack et al. [39]Another limitation is the potential for selection bias, as those patients who are more symptomatic and/or those who are not in daytime work may have been more disposed to participate, which may explain the relatively high prevalence of symptomatic patients. Future studies performing gastric mapping pre and post operatively in a longitudinal cohort would be of further interest to define the myoelectrical consequences of bariatric procedures.

The current study does not imply a causal relationship between gastric remnant degeneration and dysrhythmia and symptoms; however, the results likely have clinical implications beyond gastric bypass. In particular, the finding that a diverted stomach develops severe degeneration of the gastric conduction apparatus may become relevant in the pathophysiology and management of other medical or surgical conditions where there is prolonged stomach disuse. Relevant conditions include prolonged illness with caloric restriction, including patients on long-term nasojejunal feeding, total parenteral nutrition, and severe anorexia, or when the stomach is surgically put out of continuity. When clinically rehabilitating these patients back onto normal oral diets after a period of gut atrophy, clinicians should be cognizant that stomach pacemaker function may have become degraded, requiring additional time for recovery with return of dietary tolerance. Recovery patterns may be variable, due to the inherent plasticity and recovery capacity of the pacemaking, enteric neural, and smooth muscle components [40, 41]; however, this requires further study.

In conclusion, a third of gastric bypass patients experienced significant long-term upper GI symptoms and a reduced quality of life. These consequences are associated with severe degradation of remnant gastric electrophysiological function, although overall symptom genesis in post-bypass patients is likely to be multi-factorial.

## Supplementary Information

Below is the link to the electronic supplementary material.

Supplementary file1 (PNG 7603 KB)

## Data Availability

Individual patient data will not be available due to maintain patient privacy.
