# Supplementary figures and images for: Assessment of Gastric Remnant Activity, Symptoms, and Quality of Life Following Gastric Bypass
Source: Obes Surg. 2024 Oct 13;34(12):4490–8. doi: 10.1007/s11695-024-07534-5 (PMC11671428; doi:10.1007/s11695-024-07534-5)

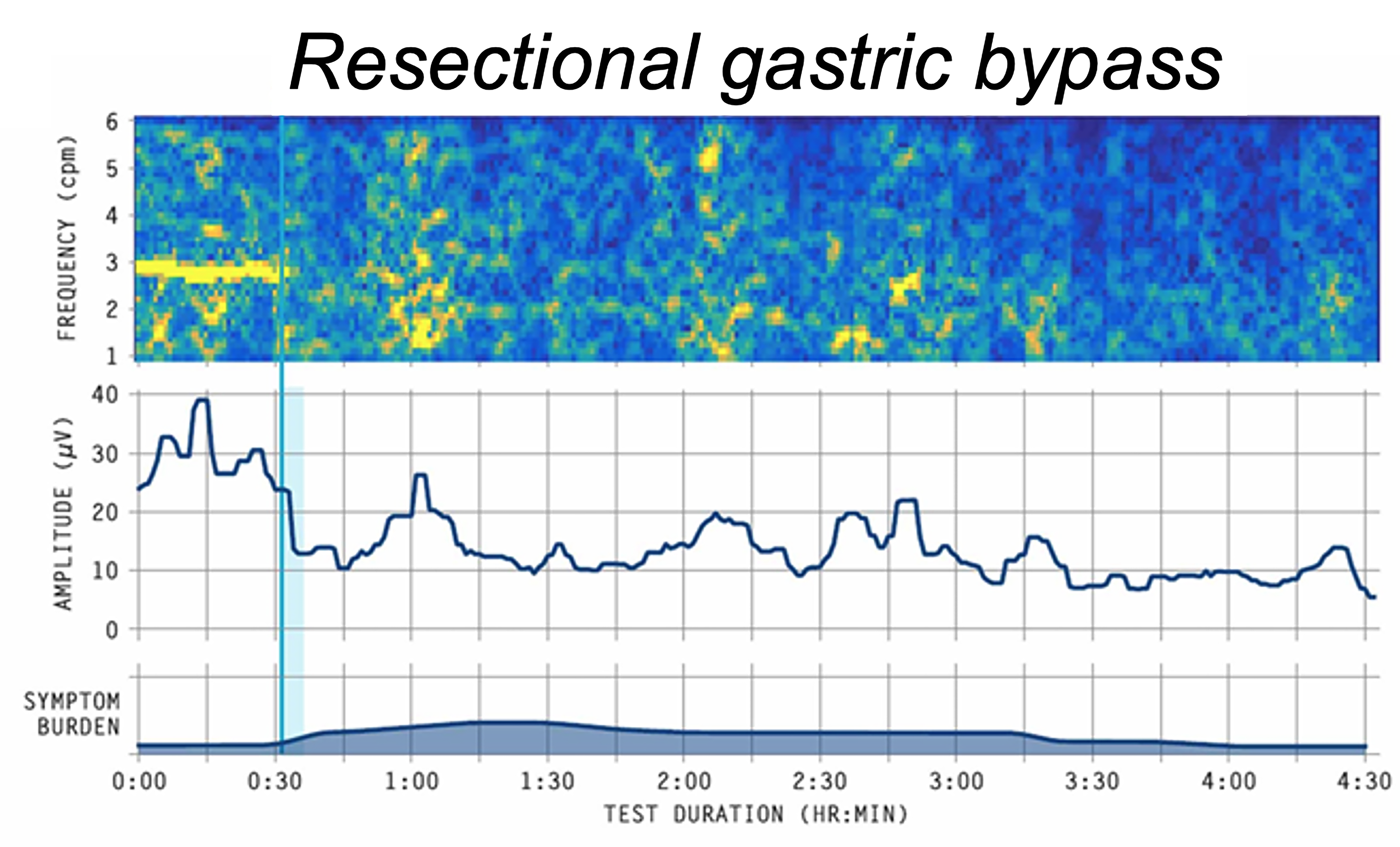

Supplement: Supplementary file 1 — Supplementary file1 (PNG 7603 KB) [file 11695_2024_7534_MOESM1_ESM.png]
